# How Regrouping Alerts in Computerized Physician Order Entry Layout Influences Physicians’ Prescription Behavior: Results of a Crossover Randomized Trial

**DOI:** 10.2196/humanfactors.5320

**Published:** 2016-06-02

**Authors:** Rolf Wipfli, Frederic Ehrler, Georges Bediang, Mireille Bétrancourt, Christian Lovis

**Affiliations:** ^1^Division of Medical Information SciencesDepartment of Radiology and Medical InformaticsUniversity Hospitals of GenevaGenevaSwitzerland; ^2^Division of ehealth and TelemedicineDepartment of Radiology and Medical InformaticsUniversity Hospitals of GenevaGenevaSwitzerland; ^3^School of Psychology and EducationFaculty of Psychology and Education SciencesUniversity of GenevaGenevaSwitzerland

**Keywords:** medical order entry systems, clinical decision support systems, adverse drug reaction reporting systems, User-Computer Interface, eye tracking

## Abstract

**Background:**

As demonstrated in several publications, low positive predictive value alerts in computerized physician order entry (CPOE) induce fatigue and may interrupt physicians unnecessarily during prescription of medication. Although it is difficult to increase the consideration of medical alerts by physician through an improvement of their predictive value, another approach consists to act on the way they are presented. The interruption management model inspired us to propose an alternative alert display strategy of regrouping the alerts in the screen layout, as a possible solution for reducing the interruption in physicians’ workflow.

**Objective:**

In this study, we compared 2 CPOE designs based on a particular alert presentation strategy: one design involved regrouping the alerts in a single place on the screen, and in the other, the alerts were located next to the triggering information. Our objective was to evaluate experimentally whether the new design led to fewer interruptions in workflow and if it affected alert handling.

**Methods:**

The 2 CPOE designs were compared in a controlled crossover randomized trial. All interactions with the system and eye movements were stored for quantitative analysis.

**Results:**

The study involved a group of 22 users consisting of physicians and medical students who solved medical scenarios containing prescription tasks. Scenario completion time was shorter when the alerts were regrouped (mean 117.29 seconds, SD 36.68) than when disseminated on the screen (mean 145.58 seconds, SD 75.07; *P*=.045). Eye tracking revealed that physicians fixated longer on alerts in the classic design (mean 119.71 seconds, SD 76.77) than in the centralized alert design (mean 70.58 seconds, SD 33.53; *P*=.001). Visual switches between prescription and alert areas, indicating interruption, were reduced with centralized alerts (mean 41.29, SD 21.26) compared with the classic design (mean 57.81, SD 35.97; *P*=.04). Prescription behavior (ie, prescription changes after alerting), however, did not change significantly between the 2 strategies of display. The After-Scenario Questionnaire (ASQ) that was filled out after each scenario showed that overall satisfaction was significantly rated lower when alerts were regrouped (mean 4.37, SD 1.23) than when displayed next to the triggering information (mean 5.32, SD 0.94; *P*=.02).

**Conclusions:**

Centralization of alerts in a table might be a way to motivate physicians to manage alerts more actively, in a meaningful way, rather than just being interrupted by them. Our study could not provide clear recommendations yet, but provides objective data through a cognitive psychological approach. Future tests should work on standardized scenarios that would enable to not only measure physicians’ behavior (visual fixations and handling of alerts) but also validate those actions using clinical criteria.

## Introduction

### Background

Clinical information systems offer integrated views on patients’ medical condition aiming at facilitating diagnosis. In order to facilitate decision making, systems increasingly not only communicate factual information but also interactively support clinical decision process. Clinical decision support systems are most often used in computerized physician order entry (CPOE) systems and typically include alerts for drug interactions. In such systems, medical alerts warn physicians when a prescription leads to a potential harmful situation for the patient’s health. Drug-related alerts can be classified into two main categories: (1) basic alerts, which verify that dosage, route of administration, and frequency of prescriptions are within the recommended range, and (2) advanced alerts, which rely on information from patients’ electronic medical records to provide personalized advice [1].

A positive effect of medical alerts on prescription behavior and, to a smaller extent, on patient outcomes can be found in the literature [2, 3]. However, research has shown that these alerts are still underutilized despite their great potential. According to a meta-study, more than half of these alerts are overridden [4]. Possible explanations for the low compliance include the lack of specificity of the alerts, their poor inclusion in the clinical workflow, and usability issues.

The human-computer interface is identified as a determining factor for improving clinical information systems (CISs) [5]. In current vendor CISs, alerts and reminders are typically displayed as pop-ups that interrupt the workflow or are displayed in the medical record using symbols to attract attention. Several guidelines [6, 7], often based on expert consensus, recommend what information has to be displayed and how this information should be conveyed. These guidelines advocate prioritization using different symbols and colors to reduce the number of alerts requiring acknowledgment and to display alerts spatially and temporally close to the triggering information. Another approach for improving usability is to reengineer the way alerts are presented and experimentally test with prototypes [8]. For example, the effect of interruptive and non-interruptive alerts on prescription behavior has been studied [9, 10].

Different approaches have been used to improve the effectiveness of medical alerts. Contextualizing the alerts [11] and eliminating those with low clinical evidence [12] or low severity [13] help to achieve a better specificity. Unfortunately, there is little consensus on what alerts are superfluous and can be removed from a system [14, 15]. Future research should aim at improving alerts’ sensitivity and specificity, better adaptation of alerts to prescribers’ personal needs, and reducing the number of alerts [16].

In our prior research [17], we learned about physicians’ use of CPOE and alerts through a work analysis of the prescription activity at the University Hospitals of Geneva. The insight obtained during interviews revealed that physicians consult medical alerts only in rare, unfamiliar medical situations, ignoring them for numerous routine prescriptions. The study demonstrated that alert handling is an active process where physicians rely on the alerting system for only complex unfamiliar medical prescriptions. This made us realize that the alert handling and the prescription of medication can be considered as two different tasks, with the former likely to unnecessarily disturb the latter.

These observations led us to propose an alternative alert presentation layout inspired by the interruption management model [18]. This model describes how interruption stimuli such as medical alerts are processed by physicians. The model shows that physicians experience cognitive load when alerts are displayed, even when they are not handled. On the basis of these ideas, we proposed a new principle advising that active alerts should be displayed regrouped in a centralized area in the prescription layout where physicians can consult and manage them. Instead of an immediate interruption, we propose a negotiated interruption where physicians are informed of alerts but can choose when to handle them.

### Study Objective

This study aimed to investigate whether centralizing alerts in a CPOE interface can lead to a reduction in the interruption of the prescription workflow without reducing the consideration of alerts by physicians.

## Methods

### Study Design

In order to compare 2 alert display strategies, 2 CPOE designs based on these principles have been compared in a crossover randomized controlled trial. In the first display design, alerts are displayed on the screen spatially proximate to the triggering information. In the second design, alerts are displayed centralized in one table. An eye-tracking device was employed for measuring inspection time on alerts and switches between alerts and prescription areas. Finally, the satisfaction questionnaire ASQ was used to measure user satisfaction with the 2 alert display strategies.

### Scenarios and Alerts

Eight scenarios aiming to solicit medical reasoning were created. The scenario contained information about fictive patient identities (name, age, and sex) and instructions for prescription of medications. Each scenario provided 2 types of alerts. Some alerts were activated and visible from the beginning. Others were triggered depending on prescriptions when following the instructions. Alerts could have 3 levels of severity (increasing from 0 to 2) and 3 levels of urgency (increasing from 0 to 2). Alerts of severity level 0 are informative alerts such as “There is already a drug of the same therapeutic class.” These alerts are relatively frequent in many situations, such as treatment of hypertension, are considered to be of “low importance,” and are thus often ignored by the physicians. Level 1 alerts are considered as severe and must usually be taken into account. However, there might be several medical reasons to overcome the alerts. For instance, “The dosage of the drug is too high considering the renal function of the patient.” Finally level 2 alerts such as “The patient is allergic (level anaphylactic) to this drug” are considered as very severe alerts, in the same group as severe interactions. These level 2 alerts should never be overpassed, except in very special situations requiring specific accesses. Severe and urgent alerts interrupted the workflow, whereas others were displayed on the screen without requiring any actions. The alerts were chosen in such a way that their different levels of severity, urgency, and modality were represented (see Multimedia Appendix 1).

Besides pharmacological alerts, physicians are confronted with other alerts and reminders during their use of the CIS. Systems warn about patients’ allergies, increased hygiene measures when patient is infected, and even reminders to consult recent patient-related data provided by another hospital division, for example, about the availability of new laboratory results. We added such alerts to our prototype to represent the full range of alerts typical for a CIS.

### Participants

Participants were recruited among physicians from the University Hospitals of Geneva and the Faculty of Medicine at the University of Geneva. Students were eligible to be included as participants when they have had some work experience as trainees in the hospital and have used the hospital’s CPOE already. The faculty of psychology of the University of Geneva approved the ethical aspects of the study that was a part of a larger PhD thesis. Because the purpose of this study was to examine the effect only on the providers, trial registration was unnecessary.

### Study Flow

#### Compared Computerized Physician Order Entry Designs

**Figure 1:**
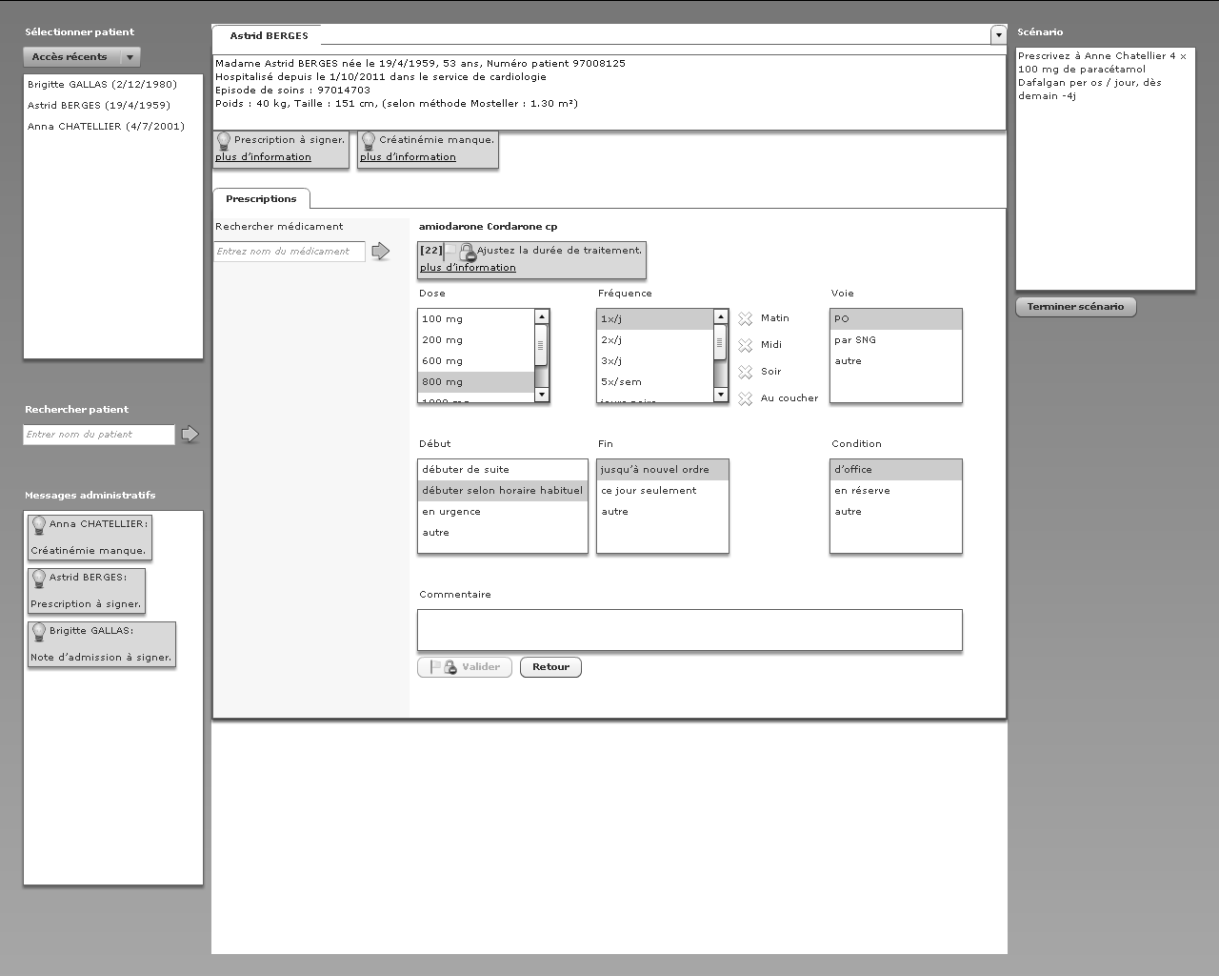
Classic computerized physician order entry design.

**Figure 2:**
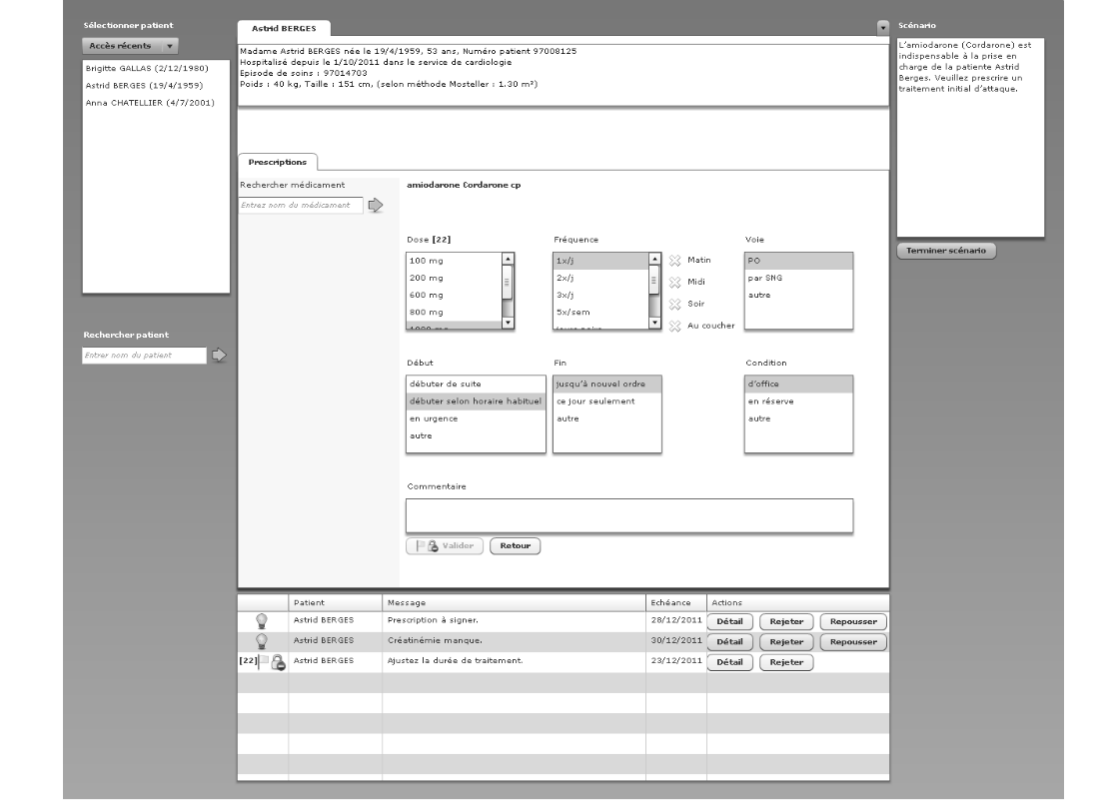
Cognitively engineered computerized physician order entry design.

To test the hypotheses, 2 CPOE layouts have been designed based on the alerts display strategy and compared in a crossover study. The 2 designs are based on a common base of the hospital’s CPOE system. In such a system (as seen in Figures 1 and 2), the column on the left allows physicians to choose the drug to prescribe. The physician can type the beginning of the name of the drug and is provided with a list of suggestions of drugs available at the hospitals. In the center of the screen, different options are available to define the dose and the frequency of administration of the drug as well as the beginning and ending dates of administration. A text area is reserved for additional comments.

#### Classic Design

In classic (CL) design (Figure 1), the *alert position* is integrated, which means it is located near the triggering information. Physicians can open an alert by clicking the link “more information.” Once opened, a detailed view is displayed in a pop-up window (not visible on the figure but located at the center of the screen) in which physicians can reject or postpone the alert. General alerts are located on the left side. Administrative alerts and reminders are on the top. Prescription-specific alerts are close to the triggering alerts. Clicking on alerts would open a pop-up with alert information.

#### Cognitively Engineered Design

In cognitively engineered (CE) design (Figure 2), the *alerts are regrouped in a defined location*. All alerts are centralized in a table at the bottom of the screen where physicians can interact with them. Three options are available to the physicians. They can click the button “detail” to open the alert in a detailed view or they can reject or postpone the alert.

### Randomization Strategy

**Figure 3:**
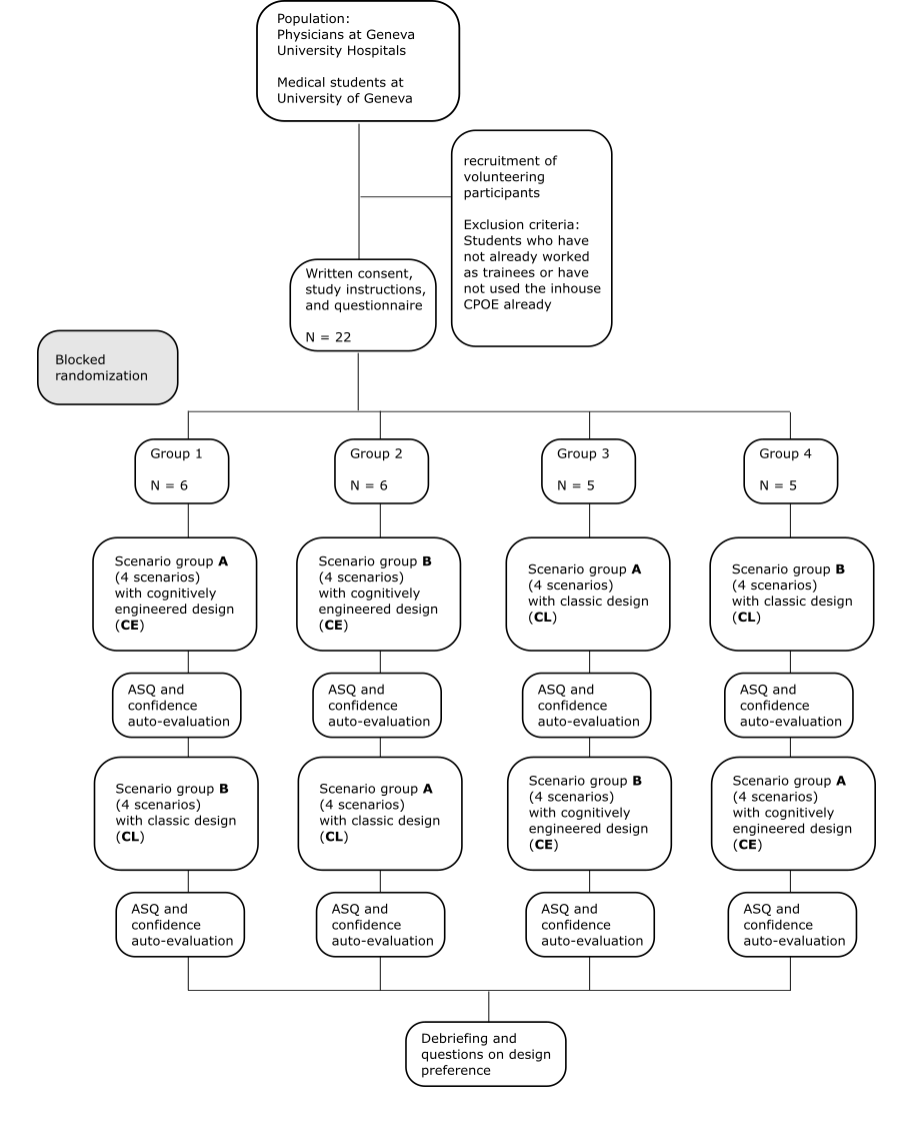
Study flow. CPOE: computerized physician order entry; ASQ: After-Scenario Questionnaire.

There is a controlled variable named *scenario group*. In each scenario group (A and B), there are 4 scenarios describing a medical case. The factors *scenario group* and *type of presentation* are randomized block wise (see Figure 3). The order of the 4 scenarios within the scenario group was not randomized, which enabled us to create scenarios using the same fictive patient twice.

Each participant performed the test individually in a dedicated test room under the supervision of an experimenter. The monitor with which the participant interacted was connected to a Tobii T120 Eye Tracker. This eye tracker is an infrared corneal reflection–based device with a data rate of 120 Hz and the accuracy of 0.5 degrees. The screen size is 17 inches (43.18 cm) with a resolution of 1280×1024 pixels. The infrared emitters and the infrared camera are integrated in the monitor. The interaction with the eye tracker is similar to that with a standard work place computer.

In a first step, the participant was briefed about the study goal and filled out a consent form. Subsequently, he or she was interviewed on his or her previous use of electronic prescription systems. Afterward, the eye tracker was calibrated to the participant’s eyes. Once the calibration was completed, the participant was instructed on how to use the interface. Subsequently, the online phase of the experiment began.

The participants started with either the CL design or the CE design. After completion of each scenario, the participants were asked by the system to what extent (in percentage) they thought they had accomplished the task. Then the participants repeated the procedure presented in this paragraph with the second design.

When the participants finished the online phase of the test, they were asked in a posttest interview about their general impression and their preference for one of the designs.

### Outcome Measures

#### Scenario Completion Time

Our primary outcome measure was scenario completion time. Scenario completion time refers to the time difference in seconds between the start of the scenario to the time they proceed to the following scenario. Participants could proceed to the next scenario whenever they considered the current scenario to be completed.

#### Prescription and Alert Handling

All interactions with the prescription prototype were captured. Every time the participant clicked a button to open, postpone, or reject alerts or to validate or sign prescriptions, a corresponding log entry was created.

#### Visual Switches

We computed the frequency of the physician’s visual focus switch between the prescription area and the different alert areas using an eye tracker. Only direct transitions from one area of interest to the other were counted. The alert areas were either the alert table in the CE design or the areas where the alerts would appear in the CL design.

#### Fixation Duration

The duration of fixations adds up participants’ visual fixations within either alert areas or prescription areas on the screen. Moreover, in the CL design the fixation duration on the pop-ups is added to the total fixation duration.

#### Satisfaction and Confidence

A user satisfaction questionnaire, the ASQ [19], was applied after each type of design. The ASQ is a 3-item questionnaire with a 7-point Likert scale ranging from *strongly agree* to *strongly disagree*. After each scenario, participants could rate the degree of confidence in the correctness of the prescription they had performed. The question was “To what degree in percent do you think that you have accomplished the task?”. Participants rated on a scale from 1 to 100 in steps of 10.

### Data Analysis

Data analysis was conducted with R statistics version 3.1.2. Shapiro-Wilk test for testing distributions for normality was used. A significance level of .05 was used for analyses. When conditions for a parametric test were met, a 2-sided paired student *t*test was used. For nonparametric tests, a paired Wilcoxon signed rank test was used. The Likert scales for the ASQ questionnaire were considered to be continuous [20]. For design preference, we used a binominal test with the assumption of a theoretical number of 10.5 supporters in each group.

## Results

### Participants

The sample consisted of 22 medical students and physicians, among whom 7 were women. Three participants were medical students, 8 were novice physicians who had their medical diploma for less than 4 years, and 11 were experienced physicians who had their medical diploma for at least 4 years. The participants had a mean experience of 2 years and 9 months with electronic prescription systems.

The physicians work in the division of general internal medicine (12), service of eHealth (2), orthopedic surgery and trauma (1), pediatric orthopedics (1), palliative medicine (1), otolaryngology (1), and medical-economic analysis (1). Concerning the physicians’ roles, there were 12 resident physicians, 3 attending physicians, 2 deputy heads of divisions, and 3 from the informatics division.

One participant had to be excluded from the analysis for perceptual and behavioral data. Only 40 % of eye-movement samples could be captured and no data were logged for interactions with the CL design for this participant. Data from the ASQ, however, were included in the analysis. The 21 remaining participants had an average of 81.86 % of eye movements recorded (SD 11.15). In Table 1, all following results are summarized.

### Scenario Completion Time

The execution time with the CL design was on average 145.58 (SD 75.07) seconds per scenario. Using the CE design, the execution was shorter with 117.29 (SD 36.68) seconds. This difference was significant with *P*=.045.

### Fixation Duration

The inferential analysis revealed a significant positive difference in the duration of fixations on medical alerts in the CL design (mean 119.71 seconds, SD 76.77) compared with the duration of fixations on medical alerts in the CE design (mean 70.58 seconds, SD 33.53; *P*=.001).

### Visual Switches

The number of switches between any of the medical alerts in the CL design and the prescription area showed that there was an average of 57.81 (SD 35.97) switches per scenario. In the CE design there were 41.29 (SD 21.26) switches per scenario between the prescription area and the table containing the medical alerts, which is significantly lower than in the CL design (*P*=.04).

### Influence of the Physician’s Experience With the Cpoe

A Kendall tau test evaluated the influence of the variables “experience with CPOE” (in months) and “medical experience group” (medical student, novice physician, expert physician) on scenario completion time, fixation duration, and visual switches. In all 3 cases, there was no significant correlation.

### Prescription and Alerts Handling

Because only 7 alerts were postponed and 15 participants never clicked on a postpone button, this variable is excluded from the analysis. Participants opened significantly more alerts in the CL design (mean 7.10, SD 4.25) than in the CE design (mean 4.35, SD 3.12; *P*=.001). Participants rejected significantly more alerts while using the CE design (mean 1.86, SD 1.39; *P*=.01) than the CL design (mean 0.67, SD 0.91).

Furthermore, we counted the number of times participants signed or validated prescriptions and added up their occurrences. The difference between the CL design (mean 8.67, SD 2.57) and the CE design (mean 7.95, SD 2.65) is not significant. The number of corrected prescriptions was counted; they include the accumulated numbers of removed pending prescriptions or stopped signed prescriptions as well as the number of times the participants removed all pending prescriptions or stopped all signed prescriptions. There was no significant difference between the CL design (mean 2.90, SD 2.17) and the CE design (mean 2.33, SD 1.96).

A test for correlations (Kendall’s tau) with the factors “experience with CPOE” (in months) and “medical experience group” (medical student, novice physician, expert physician) showed no significant correlations with the factors “number of alerts opened in CL design,” “number of alerts opened in CE design,” “number of alerts rejected in the CL design,” and “number of alerts rejected in CE design.”

**Table 1:** Overview of all results.

Performance indicators	CL^a^ design mean (SD)	CE^b^ design mean (SD)	Test and significance	
			V _df_: paired Wilcoxon signed rank test	*P*-value
Scenario completion time, seconds	145.58 (75.07)	117.29 (36.68)	V _20_= 173	*P*=.045
Fixation duration on alerts, seconds	119.71 (76.77)	70.58 (33.53)	V _20_= 204	*P*=.001
Number of switches, N	57.81 (35.97)	41.29 (21.26)	V _20_= 175	*P*=.04
Alerts opened, N	7.10 (4.25)	4.35 (3.12)	V _20_= 176	*P*=.001
Alerts rejected, N	0.67 (0.91)	1.86 (1.39)	V _20_= 13	*P*=.01
Prescriptions validated or signed, N	8.67 (2.57)	7.95 (2.65)	*t*_20_= 0.831	*P*=.42
Prescriptions corrected, N	2.90 (2.17)	2.33 (1.96)	V _20_= 92	*P*=.47
ASQ^c^ ease of use (1-7); 1 = worst, 7 = best	5.36 (1.14)	4.64 (1.53)	V _21_= 125	*P*=.08
ASQ efficiency (1-7); 1 = worst, 7 = best	5.54 (1.10)	4.64 (1.43)	V _21_= 129	*P*=.06
ASQ support (1-7); 1 = worst, 7 = best	5.04 (1.10)	3.86 (1.81)	V _21_= 146	*P*=.04
ASQ total (1-7); 1 = worst, 7 = best	5.32 (0.94)	4.37 (1.23)	V _21_= 183	*P*=.02
Confidence level (1-100); 1 = worst, 100 = best	71.97 (15.63)	66.44 (17.68)	*t*_21_= 1.65	*P*=.11

^a^CL: classic.

^b^CE: cognitively engineered.

^c^ASQ: After-Scenario Questionnaire.

### Satisfaction and Confidence

The factor “ease of use” was rated higher for the CL design (mean 5.36, SD 1.14) than for the CE design (mean 4.64, SD 1.53) but failed to reach a significant level (*P*=.08). The factor “efficiency” was rated higher for the CL design (mean 5.54, SD 1.10) than for the CE design (mean 4.64, SD 1.43; *P*=.06). This difference was not significant. The third factor “support” was rated significantly higher in the CL (mean 5.45, SD 1.10) compared with the CE design (mean 3.86, SD 1.81; *P*=.04). Finally, the “overall satisfaction” was significantly rated higher (*P*=.02) for the CL design with an average of 5.32 (SD 0.94) compared with an average of 4.37 (SD 1.23) for the CE design.

During the posttest interviews, 13 participants said they preferred the CL design and 8 said they preferred the CE design. The binomial test revealed that there was no significant difference.

Furthermore, a questionnaire evaluated whether the participants felt more confident in the solution they choose for the scenarios in either the CL or CE design. The participants had a slightly higher confidence in their solutions in the CL design (mean 71.97, SD 15.63) than in the CE design (mean 66.44, SD 17.68). The difference was not significant.

## Discussion

Our new interface design was built on the assumption that physicians get less distracted by the CE design compared with the CL design. Participants switched significantly more often between the primary task (drug prescription) and the secondary task (alert handling) in the CL design. This reduction in the number of interruptions was accompanied by significantly shorter scenario completion time.

There is no definitive answer whether the reduced attention on the alerts, measured by alert fixation duration, and the fewer times participants opened alerts in the CE design are advantageous or disadvantageous. On the one hand, it could be argued that alerts in the CE design are not seen and therefore not opened. Consequently, they do not fulfill their function of warning the physician. On the other hand, there is evidence from a prior ethnographic study [17] that physicians are not driven by these alerting systems but rather consult them in case of uncertain conditions. In this latter case, it could be argued that alerts should not divert attention more than necessary from the prescription task. This second assumption is also supported by the fact that a similar number of corrective actions have been found in the 2 designs. Therefore, even if the participants clicked on more alerts and focused their attention more often on alerts in the CL design, it had no effect on how they responded to the alerts.

Significantly fewer alerts were rejected in the CL design than in the CE design. This is probably because participants could reject the alerts directly in the CE design without opening any alert.

It could seem surprising that participants considered the CL design to be more efficient because the results proved that they were more efficient with the CE design. However, this difference between perceived time and actual time is not new [21]. Overall, the participants were more satisfied with the CL design.

An important limitation of this study is the strong similarity between the CL design and the current CIS at the University Hospitals of Geneva. Thus, physicians were used to the CL design, which might influence satisfaction ratings, alert fixation, and handling. This fact does not prevent the generalization of our findings. The cognitively engineered design presented in this study can be applied to other CPOE systems and might advocate the use of centralized alerts.

We did not evaluate whether participants looked at alerts more frequently or handled them more frequently depending on their urgency or severity. A future test could examine in detail the different types of alerts. Moreover, tests should work on standardized scenarios that would enable us to not only measure physicians’ actions (visual fixations and handling of alerts) but also validate those actions regarding clinical criteria. The alerts used in this study are conceived to be representative in their type, not in their frequency. For this reason, comparisons of our results with results from other studies reporting alert acceptance rates in a real clinical environment are not valid. Still, this study shows that the centralization of alerts influences workflow interruptions. It contradicts the proximity principle [6], which states that alerts should be close to the triggering text, but increases physicians' readiness to reject alerts that are irrelevant in their point of view.

In this work, we rely on theoretical knowledge on decision making and cognitive load to develop a new user interface for CPOE. Our formal measures, based on eye tracking, could demonstrate that following some simple design principles can affect alert handling. Centralizing alerts and making it possible to handle them in an active way reduce physicians’ workflow interruptions without modifying their prescription decisions. We did not measure the quality of the medical decision making, which should be done in a future study. When the quality standards can be met, such design principles, based on scientific measures, can be used to improve the prescription behavior and, in a future step, patient safety.

## Multimedia Appendix 1

Scenarios and alerts.

## Abbreviations

ASQ: After-Scenario Questionnaire
CE: cognitively engineered
CIS: clinical information system
CL: classic
CPOE: computerized physician order entry
