# Supplementary material for: How Regrouping Alerts in Computerized Physician Order Entry Layout Influences Physicians’ Prescription Behavior: Results of a Crossover Randomized Trial
Source: JMIR Hum Factors. 2016 Jun 2;3(1):e15. doi: 10.2196/humanfactors.5320 (PMC4911510; doi:10.2196/humanfactors.5320)
Supplement: Supplementary file 1 [file humanfactors_v3i1e15_app1.pdf]

1. Appendix: Scenarios and alerts

Scenario Nr	Instructions	Alerts	Urgency	Severity	Interaction mode
A1	Prescrivez à Anne Chatellier 4 x 100 mg de paracétamol Dafalgan per os / jour, dès demain -4j	Interaction paracétamol et métoclopramide.	1	1	Non-interrupting
		Créatinémie manque.	1	0	Non-interrupting
B1	L'amiodarone (Cordarone) est indispensable à la prise en charge de la patiente Astrid Berges. Veuillez prescrire un traitement initial d'attaque.	Prescription à signer.	1	0	Non-interrupting
		Diminuez le dosage de charge.	1	1	Interrupting
		Diminuez le dosage du traitement d'entretien.	2	1	Interrupting
		Ajustez la durée de traitement.	2	1	Interrupting
A2	Prescrivez à Brigitte Gallas un antiflatulent qui est le plus apte pour la patiente. Choissisez entre : Dimenhydrinate, siméthicone Flatulex, diphenhydramine Bonocten.	Patiente est MRSA	1	2	Non-interrupting

		Note d’admission à signer.	0	0	Non-interrupting
		Enceinte -3 mois	0	0	Non-interrupting
		Dimenhydrinate est déconseillé. [pour femmes enceintes]	1	0	Non-interrupting
B2	La patiente Caroline Ghezali, dont on sait qu’elle est épileptique, est transportée aux urgences de l’hôpital après une crise épileptique (convulsions). À son arrivée, la patiente est dans un état d’agitation. Prescrivez du clonazépam Rivotril inject.	Rivotril est déconseillé [avec un taux d’alcoolémie élévé]	0	1	Non-interrupting
A3	Vous avez un appel du médecin de garde de l’unité OH-4 par rapport au patient Cyril Muller qui souffre d’une embolie pulmonaire. Il est sous un surdosage d’héparine et il faut baisser l’héparine à 5000 UI.	Accès au dossier non authorisé.	2	1	Interrupting
		Antibiogram à ordre	1	0	Non-interrupting
B3	Carlos Fuentes est suivi depuis une semaine dans le service de cardiologie pour thrombophlébite. Ce matin, il	Accès au dossier non authorisé.	2	1	Interrupting

	se plaint de fièvre et vous devez prescrire de l'aspirine (acide acetyl salicylique) à raison de 2 cps x 3 fois par jour x 3 jours. Le patient n'est pas sous votre responsabilité.				
		Interaction aspirine et héparine.	1	0	Non-interrupting
A4	Brigitte Gallas souffre d'hypertension rénovasculaire. Veuillez prescrire du lisinopril.	Usage de lisinopril fortement déconseillé. [en cas de grossesse]	2	1	Non-interrupting
B4	Caroline Ghezali est hospitalisée dans le service de néphrologie pour un œdème aigu de poumon (OAP). Elle a une insuffisance rénale aigüe qui évolue depuis 24 heures. Prescrivez un de ces médicaments : hydrochlorothiazide Esidrex, furosémide Lasix, ou mannitol Mannitol	Esidrex est déconseillé.	1	0	Non-interrupting
		Lasix est contre-indiqué [en cas d'insuffisance rénale].	1	0	Non-interrupting
		Manitol est déconseillé [en cas d'insuffisance rénale].	1	0	Non-interrupting
